# Mentoring for Improving the Self-Esteem, Resilience, and Hope of Unaccompanied Migrant Youth in the Barcelona Metropolitan Area

**DOI:** 10.3390/ijerph18105210

**Published:** 2021-05-14

**Authors:** Xavier Alarcón, Magdalena Bobowik, Òscar Prieto-Flores

**Affiliations:** 1 School of Education and Psychology, University of Girona, Plaça Sant Domènec, 9, 17071 Girona, Spain; oscar.prieto@udg.edu; 2 Faculty of Social and Behavioural Sciences, Utrecht University, 3584 CS Utrecht, The Netherlands; magdalena@bobowik.net

**Keywords:** unaccompanied, migrant youth, mental health, mentoring, resilience, mixed methods

## Abstract

In the last few years, the number of unaccompanied youths arriving in Europe has increased steadily. During their settlement in host countries, they are exposed to a great variety of vulnerabilities, which have an impact on their mental health. This research examines the effects of participation in a mentoring programme on the psychological and educational outcomes among unaccompanied migrant youths who live in the Barcelona metropolitan area. Data in this mixed-methods study were obtained from 44 surveys with mentored (treatment group) and non-mentored (control group) male youths who had recently turned 18, as well as through thirty semi-structured interviews with mentored youths, their adult mentors, and non-mentored youths. Our findings indicated that participation in the mentoring programme improved the mentored youths’ self-esteem, resilience, and hope, as well as their desired or expected educational outcomes in this new context. We conclude that well-targeted and problem-specific mentoring programmes have positive and marked effects on unaccompanied migrant youths’ mental health. The social and political implications of these outcomes are also discussed, providing information on how interventions can offer effective networks of support for the settlement and social inclusion of unaccompanied migrant youths.

## 1. Introduction

### 1.1. Settlement and Social Inclusion of Migrant Youths in Spain

In recent years, data show that the official number of unaccompanied minors reaching Spain in small boats rose from 588 in 2016 to 7026 in 2018 [1]. This trend is not only present in Spain but also in other countries around the world. Since 2010, the number of unaccompanied minors has increased fivefold in more than eighty countries [2]. In the case of the Barcelona metropolitan area, the Catalan ombudsman reported the deficits in the current system for ensuring the settlement of unaccompanied immigrant minors and to positively favour a smooth transition to adulthood once they have turned 18 [3]. One of the main challenges young immigrants face is getting their legal residence permits processed by the relevant administration in time in order to not become undocumented, bearing in mind that turning 18 involves being left outside of the protective system that they benefitted from as minors (which includes a temporary residence permit, staying in a residential centre, and the socio-legal support of social workers and youth workers) [4]. This situation has severe consequences for their mental health and settlement process as they become invisible, homeless, and excluded from participating in the formal economy [5,6]. For those who have their permits when they are 18, the government applies a strong selection process enrolment in the transition to adulthood programme, where housing and the assistance of youth workers (mentioned by the young people of this study) is provided until they turn 21. For those who arrive as minors, the protection system helps them to reach adulthood with a residence permit that can be renewed if they have a report that positively values their integration, continue studying, or have joined the labour market [7]. Therefore, an issue in the renewal or in the processing of their documentation while they are minors, as well as the fact of them being declared of legal age by the appropriate Spanish authority upon arrival (based on forensic age estimation), makes them completely excluded from the resources that are available to the group.

Youth workers are present in the lives of young people from the moment they access the transition to adulthood programme (and the government-run flats provided) until they leave. The role of these professionals is to foster the youths’ autonomy by helping them to achieve their personal, educational, and socio-labour insertion goals. Specifically, they are asked to carry out a work plan with the young person, so that they can accomplish the goals set, and to carry out tutoring sessions in which the young person is monitored [8]. Their role is also to ensure peaceful co-existence in flats where several young people live, and also with the neighbours. However, there seems to be no explicit mention of attending to the emotional distress that their previous experiences and life in this new context might cause during settlement, a lack of intervention with these young people that has already been highlighted in the Spanish context [9].

Settlement was traditionally understood as the final stage of a migration journey, regarded as a stable social and political environment to which migrants need to adapt [10]. However, it has been emphasised that the settlement process is actually in constant flux, becoming unpredictable for migrants and capable of affecting different areas of their lives in different ways (e.g., providing stability in terms of housing and instability in their legal status) [11]. As previous research has clearly shown, the settlement of migrants is conditioned by the inclusion policies and strategies of the host country, the causes of migration, the individual characteristics of the migrant (language skills, education, employment, among others), and also the presence or absence of social support networks [12]. In this study, we have focused on seeing how this latter element can influence the settlement of young migrants, understanding that the experiences of social inclusion lived during this process, such as feeling included in a broader social environment, positively reinforce the sense of feeling socially valued, as well as of belonging and being able to participate in and contribute to the society in which they are settling [13]. This social inclusion, therefore, will also be conditioned by the ability of the young people to build bridges that connect them with the host community, which will make it easier for them feel at home in this new country [14]. Assuming that mentoring can help in the social inclusion of unaccompanied young migrants, we focus throughout this study on assessing the capacity of this intervention methodology to produce short-term effects on the psychological well-being of the youths and on how having mentors can condition their educational aspirations and expectations.

Multiple studies have indicated that the risk of suffering from mental health problems may sharpen or decrease in the new context depending on the existing public health and social policies [13,15]. In this regard, and following the principle of “in the best interest of the child”, scholars have highlighted the need to promote political measures that ensure the favourable reception and protection of unaccompanied minors and facilitate a safe transition to adulthood taking into consideration the youth’s needs [16,17]. The present study aims to demonstrate how mentoring programmes can condition unaccompanied youths’ well-being; future expectations; and, generally, their transition to adulthood in the receiving society.

### 1.2. Psychological Wellbeing and Educational Futures

In this study we focus on self-esteem, resilience, hope, and psychological distress in order to evaluate the psychological well-being of unaccompanied youths, since they are elements that can have a positive or negative impact on their mental health. Research has highlighted that a high level of well-being is a valuable resource for negotiating the settlement challenges ahead [13]. In fact, it has been recommended to nurture mentoring relationships through a programme with potential mentors to improve the adaptability of children and young people in the face of adversity [18].

Rosenberg [19] reported that self-esteem is the positive or negative reflection people have of themselves. Therefore, it involves the self-perception that people have about their failures and successes, as well as the emotional management of the negative feelings that arise. The self-esteem of young migrants has been studied because it has been shown that it correlates positively with mental health, since it buffers the negative effects of stress on depression [20,21]. Furthermore, it has been demonstrated that social support and strong self-esteem are elements that can reduce the perception of discrimination [22]. In the case of young migrants, it has also been shown that the perceived support of peers from the same age or of responsible adults (such as a teacher or mentor, in the case of our study) has a positive effect on self-esteem [23]. Additionally, it has been suggested that greater participation in a community promotes the development of self-esteem [24]. For these reasons, we consider that the participation of young migrants in a community project with responsible adults who provide them with social support will increase their self-esteem, which will have a positive effect on their settlement and social inclusion.

Resilience is defined as the capacity to access resources that nurture individual, relational, and community assets, as well as the ability to interact with others to improve this capacity through meaningful resources [25]. Therefore, these resources (provided by friends, family, or mentors) make it possible to avoid potential threats in complex situations during development. Research has highlighted the need for actions that encourage young people to be more resilient when facing stressful events that they have to deal with at this stage of their lives (i.e., the transition to adulthood) and help them to establish positive relationships with responsible adults or prosocial organisations in the new environment [26,27,28]. Masten’s resilience model [29] suggests that in order to foster resilience among migrant youths in their new context, they need to be exposed to significant risks and adapt successfully despite stressful life experiences. In this regard, some of the actions that can facilitate resilience are those that foster the quality of parent–child and mentor or teacher–child relationships in order to promote access to resources and social and human capital. However, unaccompanied migrant youths lack most of these supporting relationships once in Europe, and tend to live greater stressful experiences than other migrant youths [30] because of the lower levels of individual resources and family and social support they have. Studies that have focused on resilience have highlighted the importance of the environment in protecting against individual vulnerabilities and environmental adversity, highlighting that fostering resilience facilitates better development and psychological well-being [31]. In addition, resilience in young people has been related to better or worse adaptability to adversity [32]. Assuming that mentoring can promote the acquisition of resources to face adversity, we have considered this variable in the study, bearing in mind that greater resilience would promote better settlement and social inclusion.

Snyder et al. [33] defined hope as a cognitive set that involves the self-perceived capabilities for constructing viable paths to goals and beliefs about beginning and maintaining the route to these goals. It has been conceptualised as a positive emotional state derived from the interaction between agency aimed at achieving goals and the planning of pathways to attain them [34]. Hopeful people are seen as possessing positive thinking that reflects an optimistic and realistic perception [35], together with the belief that they can develop paths towards the desired goals [36]. Studies that have focused on hope have highlighted that it is positively correlated with life satisfaction, serving as a buffer against stressful and negative events [37]. Hopeful people perceive obstacles as a challenge to overcome and tend to show better athletic, academic, occupational, and health outcomes [36]. Additionally, high levels of hope in unaccompanied young migrants are seen to favour greater civic engagement, which is an indicator of successful social inclusion, as well as strengthening school performance and having a feeling of greater stability in their lives [38]. This favours a swift inclusion in the new community, which entails less suffering for the young person. It is due to these elements and their capacity to protect against stressful life events that multiple youth intervention projects take into account the increase in youth hope as one of the components of their action [39]. In the same vein, we consider that if mentors are able to foster hope in young people, this will translate into a more favourable process of settlement and social inclusion.

Psychological distress is a common mental health problem defined as a state of emotional suffering typically characterised by symptoms of depression and anxiety [40]. Studies that have focused on psychological distress in migrants have highlighted that it can be determined by external stressors, such as traumatic life events or the resettlement process itself [41]. It has been shown that the availability of care and the quality of support in the resettlement country can reduce the psychological distress caused by adversity [42]. However, in the case of unaccompanied youths, post-traumatic stress symptoms have been identified as being associated with reaching the age of 18, due to the revision of their legal status, which makes them more aware of the uncertainty about their right to remain in the country they are resettling in [43]. The type of residence has also been highlighted as affecting the mental health of young migrants, who report more psychological distress when they move to more independent living arrangements [44]. Taking these elements into account, we explore whether participation in the mentoring project can affect the psychological distress of these unaccompanied youths who are also in a moment of transition towards a more independent life.

Another element that is of interest for the analysis of the social inclusion of migrant youths is how they can be enrolled in education and develop educational trajectories as a process of their settlement. Previous studies approaching the incorporation of the children of immigrants in Spain and in the United States have emphasised that educational aspirations and expectations are a key determinant for future achievements in the new context [45,46]. While the educational ambitions of migrant children have been widely studied in many contexts, more research is needed for understanding the views of unaccompanied minors and youths. In this regard, we understand futures in education as “how young people see themselves in regard to the future and why futures are so valuable for them” [47]. Thus, how educational aspirations and expectations change over time and the view and vision of the young people is relevant for fostering paths for inclusion. Bearing in mind that mentoring can affect the academic achievements of young people at risk [48], we have studied whether this mentoring relationship can have any impact on the educational future of unaccompanied youths.

### 1.3. Mentoring Programmes for Unaccompanied Youths

In order to address the needs of immigrant youth, the number of mentoring programmes targeting this group have increased, especially in Europe, in the last five years after the so-called “refugee crisis” [49]. Nevertheless, there is still scarce information about the effects that the programmes specifically targeting migrant adolescents and youths have [50,51]. The meta-analyses, mostly with evidence from US programmes for general youth populations, highlight that youth mentoring interventions have a modest but significant effect on improving diverse outcomes across the behavioural, emotional, social, and academic domains [52,53]. These studies have also shown that mentoring programmes are more effective among mentored youth who have significant levels of environmental risk and among samples with greater proportions of male youths [54]. Besides, in recent years, a growing number of scholars have highlighted that mentoring, as a specific approach, can be more effective when young people are provided with the skills to recruit adults from their own networks instead of assigning participant youths to an unknown caring adult by the mentoring organisation [55,56,57]. This body of research has shown that some approaches to mentoring (such as youth-initiated mentoring, network engaged mentoring, or intentional mentoring) provide more enduring and emotionally supportive relationships than traditional approaches, because these programmes tend to empower the youths in deepening their existing ties and creating new ones [58]. However, as far as we know, there is no research showing the effects of mentoring on the mental health of unaccompanied migrant youths. What we know is that mentoring programmes can widen their social networks [59] and improve a sense of belonging and hope in the receiving society among migrant children living with their families in their new context [60].

## 2. Current Research

This study examined the effectiveness of the mentoring programme *Referents*, initiated by the *Punt de Referència* Association in 1998 [61]. The main goal of this programme is to support young people, mainly those leaving care, who, without family networks of support, start the transition to adulthood when they turn 18 after having been under the tutelage of the Generalitat de Catalunya (the Catalan government). From 2015 on, almost all participants of the programme are former unaccompanied minors who had been in the minor protection system before turning 18 and volunteer adult mentors.

The mentoring programme looks for adult volunteers who are established in Barcelona and have already completed their transition to adulthood (while also looking for young people interested in participating). After a selection process of adult volunteers and young people, training is carried out with the mentors, where the socio-legal situation of migrant youths leaving care is explained, as well as what their task as mentors involves. Each mentoring relationship (consisting of one adult and one youth) is instructed to meet once a week during a period of six months to carry out an activity. The mentoring programme practitioners suggest starting with leisure activities such as going to museums, activities in local public services, or doing sport activities. The aim of the programme is to create a bond between the mentor and mentee that facilitates significant conversations for the young person (concerns about administrative procedures, emotional discomfort, or doubts that affect their educational and occupational path), or, in other words, the provision of different types of social support.

However, despite monitoring the development of a strong and lasting bond between mentor and mentee, the *Referents* programme differs from models that provide non-specific care to their participants, in which the mentors are encouraged to provide friendship and support in general terms. These models that are less focused on solving the specific problems of the young people consider that a close relationship with the mentor is, by itself, a corrective experience that leads to a wide range of improvements in the young person’s development [62]. In the *Referents* programme, however, specific objectives for each relationship are established, thanks to exhaustive training with the mentors on the obstacles the youths need to be accompanied with through their transition to adulthood, and a strong monitoring of the relationship (with regular meetings with the mentor; the mentee; and, on some occasions, with both). It is also characterised as a well-targeted programme and is focused on solving specific problems, since the group to which it provides support is clearly defined and due to the constant coordination with the other agents that intervene in the young person’s development in order to specify what the focus of the intervention is.

The specific problems that these young people try to deal with during mentoring are usually related to learning the language, getting to know new places in Barcelona, and/or meeting new people. Here are a few of the responses of young people when we asked them why they signed up for the project:

*My first idea was that I was going to meet with someone who would be older than me, and I thought that was a great idea for me. I was going to ask lots of things about Barcelona, things about Spain, to practise Spanish … This is what I was thinking* (Amadou, mentee)

*I like that they help me from many sides. Mirela (mentor) has helped me know many places in Barcelona. […] Besides that, I have practised Spanish with her many times, and I have improved.* (Abás, mentee)

*As I said before, I felt alone in the centre and didn’t know anybody from here, from Barcelona or from Spain. I wanted to meet some kind of friend, I wanted to get to know places, practise Spanish more and everything went well.* (Hassan, mentee)

## 3. Methods

This research followed a *sequential* *explanatory* *mixed method design* which is characterised by gathering quantitative data and analysis before carrying out the qualitative fieldwork [63]. Mixed methods designs tend to provide a more complete and holistic view of the impact an intervention has rather than solely quantitative or qualitative designs. In this sense, we assessed the effects of participation in the programme on the lives of unaccompanied migrant youths in providing them with informal support in their coming of age and improving their psychological adjustment and expected or desired educational outcomes. More precisely, we were interested in how the unaccompanied youth see themselves (i.e., self-esteem), the psychological distress they experience, their future prospects (youth hope), the resilience skills they develop, how they perceive their educational near futures, and the role that social support they received has on these outcomes. In the survey data, we assessed these psychological and educational outcomes before (Time 1) and after (Time 2) participation in the programme among mentored and non-mentored (control group) youths. After the analysis of the quantitative data, we elaborated the guidelines for the interviews and carried out the qualitative fieldwork with mentored and non-mentored youths, as well as mentors.

### 3.1. Quantitative Data

#### 3.1.1. Participants

Survey data were gathered from October 2018 to October 2019, coinciding with the start date of the mentoring matches of the *Referents* programme. In this period of time, the programme began three mentoring groups, with each consisting of between 10 and 15 mentoring pairs (a mentor and mentee), since this is the number set by the programme itself to ensure correct follow-up by the mentoring expert that monitors the development of the group’s relationships. Therefore, the initial objective was to survey the maximum number of young migrant mentees, which, in this case, could have been 45. However, the programme did not reach the maximum number of recruits expected in each group and, in addition, were not all migrants (there were also Spanish youths who had left government tutelage without a family support network). Therefore, we aimed to recruit all mentees (*N* = 39); however, those surveyed in Time 1 were 32 youths (seven were discarded because mentoring matches had already started or because the youths refused to participate), and those in the control group (*N* = 26) were interviewed within one to two weeks later. From all these cases, we ended up with 21 youths in the mentoring group and 23 in the control group at Time 2, because we could not trace 12 of the pre-tested youths seven months later, and because the sample had a strong gender imbalance and thus two female participants were dropped from the analyses (initially, 91% of the mentored and 92.3% of the non-mentored youths were male). Our data confirm official statistics showing that 81.2% of unaccompanied minors in 2018 were from Morocco (see descriptive data below), and 97.7% were male [64]. The results, which include the two female participants, were consistent with the results obtained from the exclusively male sample (see Section 4).

Participants’ ages ranged from 17 to 23 years (*M* = 18.52, *SD* = 1.50) for the mentoring group and from 17 to 19 (*M* = 18.04, *SD* = 0.37) in the control group. This difference in the age ranges was because the young people in the control group were accessible, since they were in the housing resource mentioned above, which limits their stay until the age of 21, while the mentoring programme does not set an age limit in order for young people transitioning to adulthood to access it. Most of the youths had been residing in Spain for two years at the time of the study (*M* = 2.10, *SD* = 1.00 and *M* = 2.52, *SD* = 2.19 in the mentoring and control groups, respectively). The majority came from Morocco (61.9% and 82.6%, respectively), while some others came from Algeria or Sub-Saharan countries and a few from Latin America. When asked about their arrival to Spain, 47.6% of the mentored youths and 43.5% of the control group crossed the Mediterranean in a small boat, and 28.7% and 30.3%, respectively, were hidden in trucks. Most of the mentees lived in Barcelona city and a few in the Barcelona metropolitan area in shelter flats (71.4%), flats shared with other young people (14.3%), and some in a residence or in a rented shared apartment (14.3%). Most of participants in the mentoring (85.7%) and the control group (91.3%) had to move in the last year.

#### 3.1.2. Procedure

One of the main challenges of the fieldwork was to adequately select and follow, for more than six months, former unaccompanied minors between 18 and 23 years old. With this aim in mind, we counted on the active support and collaboration of Punt de Referència and the Catalan Federation of Residence Care Organizations (FEPA). Their technical staff contacted the participants, informed them about the purpose of the study, and scheduled appointments for data collection. Informed consent was gathered from all youths. In a few cases—those who were 17 at the moment of pre-assessment (Time 1)—we also asked for consent from their legal tutors (Catalan Government Agency).

All the surveys and interviews were conducted in Spanish. Language was not a barrier with most of the interviewees because the majority had a good knowledge of this language. With regard to their Spanish speaking level, at the beginning of the programme, 48% indicated that it was good, 30% very good, and 14% excellent (four participants mentioned that it was sufficient). Similarly, 41% reported that their understanding of Spanish was good, 32% very good, and 23% excellent (only two participants said that it was sufficient). Participants also reported having good overall reading and writing skills: 34% and 27% reported good, 36% and 34% very good, and 21% and 14% excellent skills, respectively. Only three had sufficient reading skills, five had sufficient writing skills, and one participant mentioned that he had some difficulties in reading and writing in Spanish We have tested a regression model where participation in the programme (yes vs. no), language ability in Spanish (i.e., the average score with the four aspects of language ability), and the interaction between the two variables were introduced as predictors and each outcome variable at T2 as a criterion variable in a separate model. We additionally controlled for the T1 scores in each model. We did not observe statistically significant interactions and thus moderation by language ability.

#### 3.1.3. Measures and Materials

We assessed diverse psychological outcomes, including psychological distress, self-esteem, resilience, and youth hope, at two time points. In addition, we evaluated the mentees’ perceptions of their educational aspirations and expectations.

- **Self-esteem.** We implemented the Rosenberg scale [19] to measure self-esteem. Participants indicated their agreement on a 4-point (completely disagree, agree, disagree, completely agree) Likert-type scale with ten statements referring to their self-image (e.g., ‘On the whole, I am satisfied with myself’; T1: α = 0.54; T2: α = 0.59).
- **Resilience.** We used a short 12-item version of the children and youth resilience measure [25]. Mentored youths were asked to respond to a series of questions about themselves, their community, and their relationships with others. They indicated the frequency with regard to these questions (e.g., ‘Do you have people around you who show interest in you?’) on a 3-point scale (yes, no, sometimes). All items were dichotomised, with ‘yes’ and ‘sometimes’ coded as 1, and ‘no’ coded as 0. We created a composite score by adding up all positive answers (T1: α = 0.66; T2: α = 0.61).
- **Youth hope.** We adapted the children and youth hope scale [33] for the migrant youths, who were asked to indicate on a 6-point scale (always, most of the time, frequently, sometimes, rarely, never) the frequency concerning six statements regarding their lives (e.g., ‘When I have a problem, I can find many ways of solving it’). This measure demonstrated satisfactory reliability (T1: α = 0.57; T2: α = 0.62).
- **Psychological distress**. We adapted the Kessler psychological distress scale [65] to our participants’ situation. Mentees indicated frequency with regard to ten questions about their psychological functioning (e.g., ‘Feel lonely’) on a 3-point scale (yes, no, sometimes). All items were dichotomised (‘yes’ and ‘sometimes’ were coded as 1, and ‘no’ coded as 0). We created a composite score for this scale by adding up the scores for the ten dichotomised items. Thus, the scale could range from 0 (when all responses were 0) to 10 (when all responses were 1, that is either ‘yes’ or ‘sometimes’). This scale showed good reliability (T1: α = 0.73; T2: α = 0.72).
- **Educational aspirations.** Participants were also asked about their educational aspirations (‘Which of the following levels of education would you like to achieve one day?’). They could choose one of eight categories, which were then dichotomised into low versus high educational aspirations. The small sample size does not allow for creating more than two categories. At Time 1, across the two groups, there were only three participants who aspired to finish a Master’s degree or a Ph.D., and only four who mentioned compulsory secondary education. In contrast, most participants (16) at Time 1 chose to finish an insertion and training programme, which is a less formal type of education (similar to the category of other courses for adults). We thus considered it logical to compare aspirations to finish courses oriented at a quick job placement with more formal forms of education, from the secondary education (which still opens up the possibility of further education) to a university degree. Three options (i.e., ‘Finish an Insertion and Training Programme (PFI)’, ‘I don’t know’, and ‘Finish some adult training course (Catalan, Spanish, others)’) were categorised as low educational aspirations, while the remaining five options (‘Finish an Intermediate vocational training diploma’, ‘Finish an Advanced vocational training diploma’, ‘Finish a university degree’, ‘Finish a Master’s degree or a Ph.D.’, and ‘Finish compulsory secondary education (ESO)’), were coded as high educational aspirations (Please note that we have decided to include the “I don’t know” option in the category of low educational aspirations in order not to lose participants who fell under this response. However, we also analysed the data excluding this category and obtained a similar result. Please see more details in the notes of Tables 3 and 4 in the Results section).
- **Educational expectations.** Participants were also asked about the level of education they think they could achieve (‘Realistically, what studies do you think you can finally achieve?’). As in the case of educational aspirations, they could choose one of eight categories, which were then dichotomised into low versus high educational expectations. Again, three options (i.e., ‘Finish an Insertion and Training Programme (PFI)’, ‘I don’t know’, and ‘Finish some adult training course (Catalan, Spanish, others)’) were categorised as low educational expectations, and the remaining five options (‘Finish an Intermediate vocational training diploma’, ‘Finish an Advanced vocational training diploma’, ‘Finish a university degree’, ‘Finish a Master’s degree or a Ph.D.’, and ‘Finish compulsory secondary education (ESO)’), as high educational expectations.

#### 3.1.4. Analytical Strategy

Quantitative data from surveys were introduced using tablets and *Qualtrics* for importing the data of the online questionnaire to SPSS format. The quantitative analysis was carried out with the SPSS Statistical Package. We used repeated measures factorial ANOVAs with participation in the programme (mentoring vs. control group) introduced as a between-subject factor, the measurement time (Time 1 vs. Time 2) introduced as a within-subject factor, and the interaction term between participation in the programme and the measurement time. A significant interaction effect would mean that the change over time is stronger/weaker in one group compared to the other, and thus that the significant change in the mentoring group is due to participation in the programme and not due to other external factors (such as simply longer time of residence in the host country). We additionally ran paired *t* tests to examine the effectiveness of the programme in the mentoring group and the control group separately in increasing/reducing (i.e., from Time 1 to Time 2) participants’ psychological distress, self-esteem, resilience, and hope.

We also applied the McNemar test to examine changes in educational aspirations and expectations. The McNemar test is used to determine if a statistically significant change in proportions has occurred on a dichotomous variable at two time points in the same population. Thus, in the present study, this test allowed us to determine the proportion of participants who had low levels of educational aspirations/expectations (a binary variable) before participation in the programme (Time 1), and who changed them to high levels of educational aspirations/expectations after the mentoring intervention (Time 2), and whether this change was statistically significant. In parallel, we tested what proportion of participants in the control group who had low levels of educational aspirations/expectations at Time 1 changed them to high levels of educational aspirations/expectations at Time 2, expecting that there would be no statistically significant change in this group. We required *p* < 0.05 as a minimal level of statistical significance.

### 3.2. Qualitative Data

#### 3.2.1. Participants

From all the surveyed youth, we selected 10 mentees, their 10 mentors, and 10 non-mentored youths using a *typical case purposive sampling* [66], and carried out thirty semi-structured interviews right after completing the T2 surveys. For the selection, we took into account youth who were not outliers in the quantitative outcomes, their ability to express more adequately their feelings and thoughts in Spanish, and their level of engagement with the mentoring experiences. In this sense, we avoided choosing those most and least engaged. For the non-mentored youth (control group), we selected those that expressed some ability to seek some assistance. We also considered similarities between the interviewees of the control group and the mentoring group, for example, their country of origin, age, place of residence, or year of arrival.

#### 3.2.2. Interview Guidelines

For the elaboration of the interview guidelines, we conducted a discussion group with four former unaccompanied minors who had participated in previous editions of the mentoring programme. They helped us to adjust the main topics of the interview to their needs and youth perspective. Mentors and mentees were interviewed individually at different times and spaces by the researchers to provide a space to freely talk about their experiences. The youths were asked about their migration journey, how they reached Barcelona, the types of support they received upon arrival and now, their stressful experiences, how they coped with them, what their aspirations and needs are, and how their mentors or other types of support had helped them in their coming-of-age process.

#### 3.2.3. Analysis

All the interviews were recorded and transcribed. We coded the materials using ATLAST.ti (Scientific Software Development GmbH, Berlin, Germany) following a flexible coding strategy [67], paying attention inductively to the information provided by the interviewees, but also taking into consideration the main categories used in the quantitative fieldwork, such as resilience, youth hope, self-esteem, and educational expectations and needs. The subthemes and codes used are presented in Table 1, as well as quote examples of every code. Other categories were also created based on what young people mentioned, which is why, in the results section, we use some quotes that do not correspond directly with the variables used in the quantitative analysis. However, all of them are related to the study’s subthemes (psychological well-being and educational futures). These categories were: *Perceived Support*, *Access to new resources, Loneliness/Isolation,* and *Planning of pathways*.

## 4. Results

### 4.1. Findings from Quantitative Survey Data

All results, including descriptive statistics, are presented in Table 2.

- **Self-esteem.** GLM repeated measures did not show any significant effects of intervention or time on self-esteem. The intervention × time interaction effect did not reach statistical significance, but the paired samples *t* tests showed that self-esteem significantly increased in the mentoring group. In contrast, there was no statistically significant effect in the control group.
- **Resilience**. We did not find any significant effects of intervention on resilience, but there was a statistically significant overall increase in resilience from T1 to T2. The paired samples *t* tests showed that resilience increased significantly in the mentoring group, but not in the control group. Yet, again, the intervention × time interaction effect did not reach statistical significance, which suggests that we cannot conclude that the effect in the mentoring group was significantly stronger than in the control group.
- **Youth hope**. No significant effects of intervention on youth hope or change from T1 to T2 were detected. There was a statistically significant intervention × time interaction effect, which confirms that the change in youth hope was significantly stronger in the mentoring group compared to the control group (and can thus be interpreted as exclusively due to participation in the mentoring programme). In line with this interaction, the paired samples *t* tests revealed that mentees showed higher levels of youth hope after the programme, whereas that was not the case for the control group.
- **Psychological distress**. We did not find statistically significant intervention, time, or interaction effects for psychological distress, and no statistically significant change in distress from T1 to T2 was detected across the two groups in the paired samples *t* tests. That is, participating in the mentoring programme did not affect the level of psychological distress of the participating youths.
- **Educational aspirations**. We were also interested in whether participation in the *Referents* programme changed the educational aims of the mentored youths. As can be seen in Table 3, the McNemar test revealed a statistically significant change in educational aims from Time 1 to Time 2 in the mentoring group. Specifically, whereas only 14.30% of the mentees maintained their lower educational aims across time, 47.60% of them changed their motivations from less (i.e., low) to more formal (i.e., high) educational outcomes, ranging from secondary to higher education degrees. Finally, 38.10% of the mentees started and maintained their formal educational outcomes, and none of the participants changed their educational aims from formal to informal. In the control group, the McNemar test was not statistically significant, indicating that educational ambitions did not change from T1 to T2. In this case, 60.8% of the participants did not change their educational aspirations, and 13.00% actually lowered them. Only 26.1% improved their aspirations.
- **Educational expectations**. In parallel, we were interested in whether being part of the *Referents* mentoring programme changed the expectations of the mentored youth with regard to the educational level they would realistically achieve in the future. As shown in Table 4, the McNemar test revealed a statistically significant effect in the mentoring group, indicating that perceived educational prospects of the mentees changed from pre-assessment (T1) to post-assessment (T2). Almost half of the mentees (47.60%) initially believed that it was only feasible for them to achieve a lower level of education, but they were more optimistic about their future education after participation. In contrast, 14.30% of the mentees maintained their lower educational expectations and 38.10% their higher educational expectations. None of the *Referents* participants lowered their educational projections. No statistically significant change in educational expectations was detected in the control group, where 65.2% of the participants maintained their educational expectations over time, and 13.00% anticipated lower educational outcome at T2 as compared to T1. Only 21.70% changed their expectations from low to high.

### 4.2. Findings from Interview Data

The qualitative results concerning the main topics of the research (psychological well-being and educational futures) are shown below. Through the analysis of the interviews carried out, we highlighted different types of social support that the youths perceived from their mentors (and that the mentors mentioned that they offered), which have had a certain impact in terms of well-being and on the decisions taken regarding what educational path to follow. In addition, we discuss the absence of certain types of support in the control group, which enabled us to understand the differences between the groups (mentored and non-mentored).

In order to suggest how the mentoring programmes can promote the acquisition of an effective support network, we highlight, in the final section of the results, how the programme guides the task of the mentors. Specifically, we focus on how the support provided by the mentors is focused on the needs of the young people due to training and the programme’s exhaustive monitoring of each relationship.

#### 4.2.1. The Role of Mentoring in Providing Emotional and Social Support

Psychological and emotional well-being is a concept that refers to aspects of psychological and behavioural functioning that involve a person’s interpersonal relationships and mental health [68]. Social support is seen as a central element of well-being in young people, being strongly associated with mental health [69] due to the perception of being cared for promoting health in a person [70]. The young mentees of this study frequently mentioned having received support in general terms from mentors and, more specifically, emotional support, which, in mentoring, is usually related to the capacity of the mentor to empathise with and listen to the mentee [71]. The case of Nordin illustrates how young people can feel better emotionally thanks to the support of these mentors. This young man, born in Morocco, explained that when he had negative feelings, he talked to his mentor, and that this mere act of talking to or meeting with the mentor had a positive effect on his emotions:

*If I don’t feel good—I’m feeling bad one day, or I’m angry—I ask him if he can meet to talk and he says yes. If I have something important, he asks me if I want to stay, no problem. He’s a really nice guy […] Because I always feel good when I am with him. He’s a good person, he treats me well.* (Nordin, mentee)

As we will see, the conversations with the mentors about aspects that worry or generate some kind of distress in the young people were recurrent. However, the mentors, thanks to a greater ability to express themselves in Spanish, were able to explain in greater depth the nature of these conversations with the young people. Below is a quote related to the social support provided by Mar (one of the mentors), who talked about one of the conversations she had with her mentee about managing negative emotions generated by adversity:

*We were talking about patience, about how difficult it is sometimes to get these things, to trust the people who are around helping him, that nobody wants him not to get them and that he knew there were a lot of people working on this, and that if he had any doubts or something was not being done properly or he wasn’t being told about, that he should ask or speak to the director of the centre … And well, I think that was important because he let it all out and I saw he was very affected by it.* (Mar, Aliou’s mentor)

Studies that have focused on young migrants have identified that perceived support, provided by adults, can improve a person’s psychological well-being and, in particular, have emphasised the positive effects it had on the self-esteem of young migrants [23]. Perceived social support was evaluated from the recipient’s perception of the availability of and satisfaction with the support provided [72]. The support from the mentors to deal with problems and overcome difficulties was mentioned as a very positive aspect of the mentoring relationship. The availability of social support by the mentors and the satisfaction of the mentees with this support were aspects that were identified in several interviews with the mentees. Below, Aliou and Dawda commented on the support they perceived from their mentors and their satisfaction with it. Dawda, furthermore, mentioned that, before his participation in the mentoring programme, such support was not available:

*If I have problems, at any time I can call her and explain my problem and, if she can, she helps me. […] This helps me because I explained this about my papers and she gave me advice.* (Aliou, mentee)

*You may have a problem and this person (the mentor) can help you fix the problem you have, and as I am not from here, the people from here know much more than I do about here, and they can tell me things that in the future can help me. […] Actually, I didn’t have an older person who I could talk about my things with in Spain, by now I have the mentor and I talk about my things with her.* (Dawda, mentee)

#### 4.2.2. Access to Social Capital

Another element that the academic literature has highlighted as favourable for the development and psychological well-being of young people is an increase in resilience [31]. Specifically, actions that favour the relationship with parents, teachers, or mentors that can increase access to resources and social capital have been recommended to facilitate resilience [18,29]. Linking relationships that connect young people with social or economic resources and that can foster greater opportunities in education, training, and work have been highlighted as important for promoting active participation in social and civic life [73,74]. These relationships result in the structures of the host society becoming more open and socially inclusive, so they are seen as key strategies to promote psychological well-being and a good settlement of the young migrants [13]. Access to new resources was mentioned by the participants in the mentoring programme. Hakim, for example, spoke to us about the different resources he accessed together with his mentor during his participation in the programme. This young mentee responded as follows when he was asked what the benefits of participating in the mentoring programme were:

*Many things … for example to be patient and to know very many things … many places. For example, a design place in Glorias, the Sagrada Familia library, Barcelona Activa (a public employment service) … To know more places, or courses, for example […] talk about things that worry me … she can also help me with these things.* (Hakim, mentee)

The mentors also talked about certain activities carried out with the mentees that could facilitate access to new resources. For example, Ariadna, Dawda’s mentor, also told us how she helped her mentee to find resources that could be important for him:

*This civic centre also has a job bank, well, an employment centre where a couple of people help you find work or make a CV … And one day I passed by and we wanted to see what they had for young people. A girl attended us and immediately … “look, here there are people who can help you make a CV, we do concerts, activities, football and many things for young people”. And yes, I took him to a place where they can really offer him the chance of broadening his social environment.* (Ariadna, Dawda’s mentor)

These resources can also help young people to better plan the pathways designed to achieve their goals in their new social environment. The creation of these connections with the mentors and with other resources of the environment can help to create feelings of hope in young people [75,76,77]. It can make them feel like they have the capacity and possibility to accomplish their future goals, since they have the agency and can create pathways to achieve them. The planning of alternative pathways that enable the creation of routes towards one’s goals is a significant component of hope [34]. In this regard, not only can the knowledge of resources be favourable, but also the conversations with the mentors that enabled the young people to reflect on their pathways were important during the mentoring. Amadou explained that, for him, it was important to be in contact with other people, because, in that way, he could share ideas related to his own career. Moreover, participating in these types of projects helped him to feel more relaxed, which allowed him to plan his future under less pressure. After being asked why he decided to participate in this mentoring programme, he responded as follows:

*Because I like having relationships with a lot of people. Because a memory is a memory, but your memory and my memory, if we work together, there will be two ideas that are worked on. If it’s only my idea, I can’t do anything. […] Well, since I came here with many projects, collaborating with them, I began to forget my stuff, I began to relax with my stuff …* (Amadou, mentee)

#### 4.2.3. Promoting the Mentees’ Interest in Formal Educational Paths

The social support that mentoring can provide in terms of advice can have an effect not only on the level of hope and psychological well-being of the youths, but could also have an effect in terms of their educational futures. Previous studies with young migrants have highlighted that educational aspirations and expectations are fundamental for accomplishing futures goals in their new context [45,46]. Self-defined paths for young people are usually based on the need to find a job in order to be independent in the new setting and not dependent on the support of philanthropic or care organisations. In addition, the added pressure of wanting to help their family drives them to choose educational paths with quick access to the labour market. The case of Hassan illustrates that, if conditions are optimal to be able to continue studying, it is easier for the youths to make this decision, since, in this way, they can acquire a professional path that allows them to access better paid jobs:

*It depends. I want to work and help my family a little and myself. If I am fine here, I would like to continue studying. […] I want to get an 8.7 to do vocational education and training [...]. Cooking, hairdressing … Then the higher education as well, if I can. And continue studying.* (Hassan, mentee)

Once these conditions are met, the difficulty is in the choice of career path that fits their interests, or understanding the differences between different educational levels, among others. This is where the mentor can provide assistance, promoting the young person’s interest in educational paths that enable them to achieve higher levels of education. Hakim explained that, thanks to his mentor, he understood what path he could follow after completing the PFI, instead of entering the job market, as well as explaining the disorientation he felt regarding his educational future:

*Because when I wanted to do the first course, I did a course in waitering and didn’t know what courses there were … you know? I did a course for work as a waiter and as a cook because everybody does that […] She (the mentor) explained to me that, for example, if you don’t not have ESO, you can study a PFI to do an Intermediate vocational training diploma, and when you pass the Intermediate vocational training you can do the Advanced vocational training diploma and then, if you want, you can go to university.* (Hakim, mentee)

We observed that the more informal conversations with mentors could help consolidate a more prolonged educational path within formal education. In this regard, Antonia (Hassan’s mentor) and Elisabeth (Hakim’s mentor), explained what these conversations were like:

*Poor thing … I think he is very lost … and the fact that he told me “I want to work, I want to work” and that I told him to take advantage of the time now and study … it could be that saying to him “don’t worry now about money, you could be some time without work …”, but of course, I suppose that also behind this he feels the pressure of “I have to comply”.* (Antonia, Hassan’s mentor)

*Sure, he did the waitering course… the practical training… this and that, but then he was going to do a cooking course, but in the end he was going to do one in maintenance … but he wants to do English … he is very disoriented. […] We went to a place with technological stuff because he really likes everything related to computers and they give free courses on all things related to technology and you can sign up …* (Elisabeth, Hakim’s mentor)

#### 4.2.4. Lack of Social and Emotional Support in Non-Mentored Youths

Previous studies that have focused on young people in care and leaving care have emphasised that the professionals that work alongside them are usually seen by the youths as representatives of a formal and instrumentalised world. Specifically, they see them as people who do their job, focusing on the solution of specific problems [78]. Furthermore, in the Spanish context, it has been highlighted that there are few resources or services that accompany the young migrants in attending to the emotional distress that settlement may cause [9]. In this regard, the non-mentored youths in this study emphasised being able to talk about some of their emotional discomfort with the youth worker of their flat. However, these conversations were limited to formal spaces and specific tutoring sessions in which the youths were summoned at a specific time of the week to talk about various aspects of their work plan during their stay in the flat of the transition to adulthood programme. Arturo explained the following to us when we asked him about his concerns and whether he talked about them with somebody close to him:

*I don’t usually talk about my problems, but one person like that … is Neus (youth worker). I sometimes have tutoring and we talk about how we are and stuff, and some discomfort comes out and we talk about it.* (Arturo, non-mentored youth)

This contrasts with the role that a mentor can play in the life of a young person, since the former takes time to be present in the life of the latter without being restricted to a specific time and space during the week, being seen as a result as someone from a more informal world [79]. The academic literature has highlighted that the informal nature of the mentoring relationships facilitates the appearance of emotional support or advice support because it allows them to arise in the context of a normal everyday conversation [71]. That is probably why it was difficult to identify the perceived social support of the non-mentored youths, who were explicit and even mentioned that sometimes it was difficult to find the support they needed:

*Sometimes, if I have a serious problem, I don’t know what to do. […] Well, I don’t know, that nobody helps you here; well, difficult.* (Mourad, non-mentored youth)

The availability and quality of the forms of support in the country in which a person is settling has been shown to have an impact on psychological distress [42]. Furthermore, support networks are important for reducing the feelings of isolation and loneliness in migrants who are settling in a new country [50,60]. Loneliness in unaccompanied young migrants appears in the absence of people who care about them, and it has been demonstrated that new social contacts have a positive effect in combatting it [80]. However, in this study, we have found that young migrants who did not participate in the mentoring programme lacked strong networks of support and new social contacts. In fact, feelings of loneliness and the lack of forms of support were aspects that the non-mentored youths mentioned in the interviews. In this regard, Mustafá, perhaps because he was not able to connect with anyone who could help him to solve his problems, expressed the following:

*Everything that you have to do, there is nobody that’s going to help you. You always have to do things on your own, with the language or without the language. Nobody cares about your stuff; you have to do it alone. Before, in the centre, they always said “come on, I’ll go with you to the doctor”, “come on, we’ll go with you to such and such …”, “we’ll go with you to look for courses.” Always, everything that you’re going to do, “we’ll go with you.” Here they don’t go with you, they say: “Ok, go alone, you’re an adult”, but this is normal, it doesn’t matter. […] When you are in Morocco, you always share things with your family, your parents … and now you live here alone. You’ll always be alone.* (Mustafá, non-mentored)

It has been identified that turning 18 generates a degree of psychological distress in unaccompanied youths [43]. In fact, this distress was identified in the two groups that were studied (mentored and non-mentored youths). However, independent life after leaving the residential centre they had stayed in as minors seemed to affect the non-mentored youths more. Just as the mentored youths were able to identify people around them (mostly the mentors) who gave them support, the non-mentored youths highlighted this feeling of loneliness after moving to the flat of the transition to adulthood programme. This more independent life has been shown to generate greater psychological distress in their lives [46]. Rashid explicitly mentioned this greater difficulty in finding support after turning 18 and moving to the transition to adulthood flat:

*Now since I am over 18, I have some difficulties. You have to get by on your own. You have to make a living by yourself. Nobody helps you. […] It’s not like being a minor. When you are older you have to do everything alone, nobody helps you. If you want to do something, manage papers or go to an office, you have to learn to speak, learn how to do it. The difference when you are younger is that in the care centre they do everything for you.* (Rashid, 19)

#### 4.2.5. Absence of Alternative Educational Pathways in Non-Mentored Youths

The lack of social support also seems to have had an effect on the creation of pathways designed to continue studying at higher levels of formal education. As we mentioned, these young people have doubts about whether to continue studying in formal education or whether to seek courses that guarantee rapid access to the labour market. Ahmed, another young man from the control group, also expressed many doubts about what to study. These doubts have made him undertake a training and insertion programme to be a sales assistant, a course in the hotel sector, as well as various adult training courses. As he commented, the youth worker told him that he had to study before working, and he promised to do so, in part, to be able to access the housing benefit. However, his explanation lacked any positive mention of continuing studying at higher educational levels, such as in the case of most of the young people in the control group:

*I live in a government-run flat, and when we had to enter it we had to sign some rules. These rules say that you have to study. Moreover, when we have to change the papers, we need studies, if not, they can take them away from us. So we are now studying and obtaining diplomas so that when we have the papers, they authorise us to work and we can work in many places. […] I have 4 or 5 professional diplomas: in sales, I am also studying in the hotel sector and I also want to have a diploma for hairdressing. Because if one day there is no work in sales, it won’t matter, we can go to the hotel sector. If there is no work in the hotel sector, then we’ll try hairdressing*. (Ahmed, 18)

This strong wish to enter the labour market drove Ahmed to follow an educational path that was more focused on gaining quick labour insertion. Generally speaking, the possibility of studying higher education courses within formal education was not mentioned, nor did they mention having received messages that promoted doing higher educational studies or having had meaningful conversations with adults in their social environment that encouraged reflection on their future education. Other young people of the control group mentioned the possibility of continuing to study some courses in the afternoons, after finding a job. In this regard, another of the non-mentored youths, Youssef, made a similar reflection to that of Ahmed with regard to the aim of gaining quick labour insertion. He explained why he preferred to continue with the training course that he was doing, instead of seeking a more prolonged path in formal education:

*I am doing a PFI (training course) in cooking, waitering and catering in general, in which I work as a waiter and cook. […] Next year, you choose only the thing you like most, I mean, if you want to be a waiter or a cook. I have considered it, but I don’t know if I’m going to continue with one of cooking or waitering. Also, [the study centre] hires you if you do well. If you look for work, they help you get it, and they also hire you. So in this way I’m not going spend two years for nothing.* (Youssef, 18)

Thus, taking these qualitative analysis findings into account, we suggest that in order for young people to develop this motivation to continue within formal education, it is important that they receive messages that help them reflect on this option of continuing to study at higher levels of formal education.

#### 4.2.6. Targeted and Problem-Specific Mentoring Programs for Unaccompanied Youths

A programme is considered to have a targeted approach when mentoring is directed specifically at a young population and when it is designed specifically to address the challenges of this population [64]. Studies that have focused on examining the different approaches to mentoring have highlighted that programmes that focus on the challenges of a specific population have a greater effect in terms of academic [81], psychological [82], and social [83] outcomes. One important element here is that mentors are trained so they can directly address the problems related to the group of young people they are trying to support [83]. The mentors of the *Referents* programme highlighted this training as very important in helping them feel equipped and able to better understand the difficulties of the young people. Here are some comments from the mentors during the interviews. They mainly highlight as positive elements of the training regarding the possible difficult situations that may arise during mentoring and learning the legal context of the young people:

*In the course, at the beginning, they told us (mentors) everything that might happen to them (mentees) […] It of course puts you in a difficult situation and I think that is very good because it’s a way to make you understand that not everything is beautiful. Perhaps the boy comes to you one day and asks you something … and you don’t know how to respond …* (Mirela, mentor)

*When you start training you don’t quite understand why you have to do a training… But then you find yourself in so many situations … And you think: “they told me that this would happen and that the other thing would happen too …”.* (Antonia, mentor)

*They gave us the context of the current legal framework of the youths that arrive, whether they have asked for political asylum or with regards to unaccompanied minors … they tell you all about this.* (Inés, mentor)

The mentors also emphasised that the support of the mentoring programme was constant throughout the established mentoring period. Therefore, any doubts that arose regarding how to act were also addressed by the programme’s supervising team. This made it easier for the mentors to know what to do and how to handle doubts practically the moment they arose:

*I think the feedback of Helena (mentoring programme practitioner) was … We were able to speak on many occasions; I left her a lot of audios (via instant messaging), especially at the beginning about how the session (the meeting with the young person) had gone … The answers help you a little to resolve concerns and doubts that the sessions brought up and other more specific things.* (Miquel, mentor)

*Especially on the issue of persevering more or less with their education. For me, education is the way out of marginality, to the extent that this is possible for each person … Anything that is education is the best that a person can do … And someone of this age (talking about the mentored youths) I am very sure about this: education, education, education. The thing is that for them a work permit is important. So, of course, I asked him (the mentoring programme practitioner) if I should insist […] How far should I press here … things like that … A few guidelines to avoid putting my foot in it.* (Ariadna, mentor)

## 5. Discussion

This research aimed to identify whether the absence or presence of adult mentors providing social support can condition unaccompanied youths’ well-being and their future prospects in their new context, especially taking into account implications for their transition to adulthood. The findings of this study showed the existing connections between the social support unaccompanied youth have in the receiving society, their mental health, and the possibilities for constructing new educational futures. Those who have less caring relationships (such as those from the control group) counted on the support of youth workers who helped them to comply with the formalities of their transition to adulthood, but most of them felt left emotionally on their own. Thus, coming of age for these young people became an odyssey that altered their mental health and well-being due to the pressure they felt when coping with housing and legal status once they turned 18 and left the minor protection system. However, we have observed that those youths who had broader social support because of their participation in a mentoring programme saw improvements in their psychological well-being outcomes (such as self-esteem, resilience and youth hope), and that such support provided them with the emotional stability to seek a higher educational path and achieve a safer transition to adulthood.

These results obtained with unaccompanied migrant youths corroborate prior research showing the significant effects of mentoring programmes on various youth outcomes, such as resilience, self-esteem, or youth hope, for youths either in care or transitioning out of the foster care system [84,85]. While some meta-analyses have shown that these effects may be modest for youth mentoring programmes in general (for example Hedges’ *g* = 0.21; both in Raposa et al. [52] and DuBois et al. [54]), the effect sizes of programmes which have a clear targeted population and are more problem-specific (such as the one we studied) tend to be higher, and double those of programmes with non-specific approaches [62,86]. The evidence from this study supports this argument because the effect sizes for the values studied are well above 0.50.

More specifically, with unaccompanied migrant minors, other inquiries have stressed that mentoring encourages young migrants’ hope and feeling of belonging [60], as well as perceptions of the social support they have in the host culture [59]. This study further contributes to this field by shedding some light on the effects on unaccompanied youth once they turn 18, considering that our quantitative discoveries showed large effect sizes (‘d’ around 0.8) for the mentoring group on resilience (0.7), educational aspirations (0.86), and educational expectations (0.86), or medium effect sizes (‘d’ around 0.5) for self-esteem (0.54) and youth hope (0.45). However, we could not identify significant differences in psychological distress, probably because this variable needs a more professionalised intervention to show significant changes.

Our qualitative findings showed that the mentor’s availability to be present in complex situations in which youths are involved during the transition to adulthood and becoming settled explains the differences in the quantitative results between the treatment and control groups. The mentoring relationship makes it easier for the young people to share feelings that undermine their self-esteem, which can help them to create a more positive impression of themselves thanks to the conversations with the mentors.

Furthermore, mentoring relationships help youths to assess their needs by becoming an important ecological resource for their resilience and strength [87]. Mentoring is an opportunity for them to share ideas about their own trajectory with people who can guide them in making decisions or even to improve individual and relational resources to deal with stressful and complex events. In the same way, mentors promote a more positive vision in young people about their plans, since the messages of being patient and encouraging help the mentees to be more hopeful about their future. Therefore, all of this social support also promotes higher educational expectations and aspirations, since this psychological well-being makes it easier for them to have a positive impression about what they can achieve in their lives. Furthermore, mentors establish conversations about how to continue with their educational path, being guided by a person who knows how the formal educational system works and who can help to specify their self-defined educational plans.

We can therefore see how mentors are able to provide a wide range of types of support, thanks to an appropriate orientation by the programme in the goals to be addressed with the young person. The mentoring practitioners that monitor each relationship knew how to work individually with the young person and with the mentor regarding the goals that were proposed in the relationship, which bore fruit in the different realities of each one. Those young people who needed to be introduced into new environments or to solve certain procedures were provided with instrumental support, such as concrete or companionship support. The young people who needed certain recommendations to continue on their educational path or who needed to hear suggestions from somebody with more experience than them received advice support. Additionally, those who needed someone to understand or value them in difficult moments were provided with emotional or esteem support. In addition, it is important to emphasise that this emotional support was fairly common in all the mentoring relationships.

We also highlight how the mentoring programme supervises the mentors while the mentoring relationship lasts. An important element for the mentors was the training and the issues addressed therein. They emphasised that it was useful for their work as mentors to receive information about the socio-legal situation of the youths, as well as the ability of the programme to make them aware of the difficulties they may encounter during the relationship. They also highlighted as very positive the accessibility they had to the mentoring programme practitioners throughout the relationship, since they could resolve doubts and concerns in a very direct and simple way. This made it easier for the mentors to better assess the type of support they were giving the mentees and to better understand where this support should be focused. We believe that this is a relevant finding for determining how and why mentoring interventions with unaccompanied youths can be effective in providing new support networks. We therefore suggest that the results in terms of psychological well-being and educational futures need to be contextualised within these elements highlighted by the programme mentors (a well-established training and a supervision or monitoring that guides the mentors with their doubts).

In contrast, the interviews with the non-mentored youths showed an absence of adults responding to their emotional needs. Youth workers are present to provide types of support that are very focused on specific problems, but they are not present in some circumstances in which youths need emotional support. This is something that we can observe in the quotes of the young people of the control group when they explain that they have to deal with many of their problems alone because they do not have anybody to count on, or when they explain that they sometimes do not know very well who to talk to about their problems. Youth workers can solve some of the young people’s difficulties, but rather are mostly focused on specific needs related to their residence or work permits and on the search for courses that guarantee them a quick job placement. This lack of support promotes the feelings of isolation and loneliness that were evident during the interviews, which makes it difficult for youths to develop positive self-esteem for use in their daily lives, to be more resilient with complex situations, or to have hope in a promising future. Moreover, they also show the existing and absent dialogues unaccompanied youth have with their caregivers, and how the existing social categorisation and expectations of them may be challenged with a more holistic assessment connected to their hopes and perspective.

## 6. Limitations and Future Research

This research is not devoid of limitations. It is relevant to highlight that those who participated in the fieldwork are youth enrolled in the Catalan residential programme for former youth in care. They do not represent all unaccompanied youth because a relatively high number of unaccompanied youths are not enrolled in that programme due to the few available places. Unfortunately, a significant number of them also become irregular when they turn 18 because their residence permits were either not applied for or not renewed by the relevant entity. Nor could we access as many female participants as we would have liked because of the lack of girls in the *Catalan Federation of Residence Care Organisations*. Furthermore, there are fewer unaccompanied girls that migrate, and those that accomplished their migration journey suffer other aggregated vulnerabilities that complicate access to them. For these reasons, we cannot have an accurate vision of youth needs and difficulties disaggregated by gender.

Another limitation of this longitudinal research is the number of study participants and the difficulty in carrying out a follow-up of such a vulnerable population. The small size of our sample also limited the power of quantitative statistical analyses. Sample size may have limited the possibility of reaching significance in some of the findings obtained in this study, especially with repeated measures ANOVAs. Specifically, although we observed a statistically significant increase in self-esteem and resilience in the mentoring group and no such change in the control group, we did not find significant interaction effects with these variables. This suggests that this change could be still due to some external factors such as length of residence or housing. The legal situation of the young people can be very varied. There are young people who quickly receive a job offer and process a work permit, others can spend months waiting for the resolution of their residence permit, others have received the residency permit shortly before entering the transition to adulthood programme, and there are those whose permit has expired and have to renew it. All of these situations probably have a different impact on the psychological well-being of the youths, and it is very difficult to control them. Similarly, the length of time spent in the flats of the transition to adulthood programme can be an external factor that is difficult to control. There can be young people that are starting their stay in the flat, others who are ending it, and also within this latter group there will be differences between those who have found another housing resource for when they turn 21, those that have obtained a job offer and will now be able to live independently, and those faced with the possibility of being left without housing and cannot afford to rent a room for themselves.

Nevertheless, one needs to consider the reality of mentoring programmes for unaccompanied migrant youths: they usually involve a smaller number of youths, and thus, it is difficult to count on more robust samples. It is also important to highlight that we did observe statistically significant effects even with a limited sample with simpler statistical procedures (paired samples *t* tests) in all except for one outcome (psychological distress), and thus, it is reasonable to assume that these effects would have held with a larger sample. Our study should also be replicated with more reliable measures. Several instruments in our study showed a relatively low reliability, which might have been due to language difficulties among some participants. Thus, future research should ensure participants the possibility to respond to measures in their native language. Finally, our aim was not to provide outcomes to generalise over other contexts. Rather, we aimed to show how social mentoring interventions have relevant implications for the social inclusion and well-being of unaccompanied migrant youths.

Finally, we would like to highlight some recommendations for future research aiming to improve the well-being and health of unaccompanied youths in their coming of age. First, longitudinal studies with follow-up measurements need to be conducted in order to test the long-term effects of these mentoring programmes. In this work, we have identified significant effects of involvement in the programme on these young men’s mental health and educational outcomes. However, future research is needed to determine whether these effects would continue, increase, or decrease over time, and whether mentoring relationships can last beyond the time stipulated by the programme. Secondly, future studies should aim to replicate the results obtained in this research in a larger sample of unaccompanied migrant youths, and to further disentangle the complexities of their support networks and the implications they have for their well-being and the construction of these young individuals’ life trajectories. Finally, given that gender differences could not be explored in this research due to the absence of a necessary number of unaccompanied girls among the participants, there is also a pressing need to delve into the possible differential effects of social mentoring programmes among boys and girls.

We also consider that the findings from this research have relevant political and social implications for the social inclusion of unaccompanied youths. Our results speak to the lives of thousands of migrant youths who face the challenge of migrating to Europe on their own and lacking social support in this new context. Our findings suggest the benefits of mentoring programmes for their settlement, resilience, and well-being, and there is a pressing need to invest more public funding into fostering their social inclusion and a safer transition to adulthood. It is worth noticing that new approaches to youth mentoring (such as youth-initiated mentoring, mentioned earlier) are difficult to implement with unaccompanied youths due to the lack of pre-existing networks of support, family ties, or informal mentors in this new context. Thus, this research stresses that some approaches to mentoring, such as the one analysed, which also aims to increase the availability of caring adults for migrant youth, are critical and favour factors that promote well-being among unaccompanied migrant youths.

Moreover, it is also important to highlight that mentoring interventions also need to go hand in hand with structural changes on immigration policy in order to favour access to citizenship and avoid transitions to “illegality” when the youths officially become adults [5]. Further, interventions need to go beyond the provision of basic assistance and protection. It is necessary, as Chase [88] suggests, to offer these young people supportive relationships so that they can build their most immediate future based on fulfilling their capabilities and well-being.

## Figures and Tables

**Table 1:** Subthemes, codes, and quote examples.

Subthemes	Codes	Quotes
Psychological Wellbeing	Self-Esteem	I was angry for a few days because I didn’t understand. There were some things that for me were difficult to understand and she said to me: “Let’s see, you’ve been here for a year and you understand Spanish. If I went to Morocco and I stayed there 2 or 3 years, I wouldn’t learn it like you”, then I relax and I think I’m speaking well. (mentored)
	Resilience	I found some things difficult and I felt a bit embarrassed and a bit sad, but in the end I understand it a little and I have seen that I have to force myself to speak, because if I don’t speak I won’t learn anything. From that moment [to] now, I always have the courage to study things. Even Spanish and Catalan, but not only that, I want to study in my life until the end. Because life is a study class. (mentored)
	Youth Hope	Yes, sometimes it worries me. Because if they take away your NIE (tax identification number for foreign residents) you have no papers or anything. What are you going to do? Nothing, you’d be better going back to Morocco. […] I would feel a bit like I hadn’t finished what I wanted to do. I would feel a bit like something is lacking. I won’t have reached the future, that’s what I mean. (non-mentored)
Educational Futures	Expectations	I want to get an Advanced vocational training diploma, the problem is that I don’t have a work permit. […] I am in a foundation that pays for the rent and everything, but you can’t be with a foundation for more than 4 years and it will take more than 4 years to get an Advanced vocational training diploma […] So, I only have 2 years left in this foundation. In 2 years I’ll get the Intermediate vocational training diploma. (mentored)
	Aspirations	Well, continue, because I already have the PFI (Insertion and Training Programme) and I have the letter of recommendation from a shop. I also have the language and everything. I want to continue with these hotel and catering courses … (non-mentored)

**Table 2:** Participation in the mentoring program and well-being outcomes: descriptive statistics, repeated measures ANOVA with between-subject (group) effect, and paired *t*-tests per group.

										Comparison			
Variable	Group	Time 1 *M* (*SD*)		Time 2 *M* (*SD*)		*CI*	*t*	*p*	*d*	Test ^1^	*F*_(1, 42)_	*p*	η²
Self-esteem	Mentoring	2.87	0.23	3.01	0.30	[−0.27; −0.02]	−2.41	0.026	0.55	1	1.03	0.316	0.02
										2	3.16	0.083	0.07
	Control	2.86	0.33	2.86	0.30	[−0.12; 0.12]	−0.08	0.941	0	3	2.80	0.102	0.06
			
Resilience	Mentoring	9.95	1.40	10.76	0.44	[−1.40; −0.22]	−2.88	0.009	0.74	1	1.24	0.273	0.03
										2	4.94	0.032	0.11
	Control	9.96	1.52	10.04	1.33	[−0.68; 0.51]	−0.30	0.765	0.05	3	3.21	0.081	0.07
			
Youth hope	Mentoring	5.10	0.73	5.52	0.60	[−0.81; −0.04]	−2.33	0.031	0.58	1	0.04	0.846	0.001
										2	0.94	0.339	0.02
	Control	5.45	0.83	5.25	0.82	[−0.10;0.51]	1.39	0.179	−0.22	3	7.33	0.010	0.15
			
Psychological distress	Mentoring	4.95	2.64	4.76	2.84	[−0.61; 0.99]	0.50	0.623	−0.09	1	0.08	0.779	0.002
										2	0.01	0.964	0
	Control	4.96	2.69	5.17	2.37	[−1.14; 0.70]	−0.49	0.630	0.11	3	0.48	0.494	0.01
			

*Note.* Mentoring group: *n* = 21, control group: *n* = 23. *M* and *SD* represent means and standard deviations, respectively. ^1^ ANOVA contrasts in the following order: 1 = group’s effects; 2 = time’ effects; and 3 = interaction effects (group * time). To calculate Cohen’s *d*, we used a procedure described in Morris and De Shon (2002, p. 111), who suggest estimating the effect size for single-group pre-test–post-test designs by taking the correlation between the pre- and post-test into account. Statistically significant effects are in bold and marginally significant ones are in italics.

**Table 3:** Participation in the mentoring program and educational aspirations: the McNemar test.

Variable	Group		Low Educational Aspirations (Time 2)		High Educational Aspirations (Time 2)		*p*
			*f*	%	*f*	%	
Educational aspirations	Mentoring	Low educational aspirations (Time 1)	3	14.30%	10	47.60%	0.002
		High educational aspirations (Time 1)	0	0.00%	8	38.10%	
	Control	Low educational aspirations (Time 1)	7	30.4%	6	26.1%	0.508
		High educational aspirations (Time 1)	3	13.00%	7	30.4%	

*Note.* We repeated these analyses excluding participants who responded “I don’t know” to the question about educational aspirations. In this case, the McNemar test was also statistically significant in the mentoring group (*p* = 0.016), whereas it was non-significant in the control group (*p* = 0.453), with 18 participants in each group.

**Table 4:** Participation in the mentoring program and educational expectations: McNemar test.

Variable	Group		Low Educational Expectations (Time 2)		High Educational Expectations (Time 2)		*p*
			*f*	%	*f*	%	
Educational expectations	Mentoring	Low educational expectations (Time 1)	3	14.30%	10	47.60%	0.002
		High educational expectations (Time 1)	0	0.00%	8	38.10%	
	Control	Low educational expectations (Time 1)	11	47.80%	5	21.70%	0.727
		High educational expectations (Time 1)	3	13.00%	4	17.40%	

*Note.* We repeated these analyses excluding participants who responded “I don’t know” to the question about educational expectations. In this case, the McNemar test was also statistically significant in the mentoring group (*p* = 0.016), whereas it was non-significant in the control group (*p* = 0.453), with 18 participants in each group.

## Data Availability

The data presented in this study are openly available in Harvard Dataserve repository at https://doi.org/10.7910/DVN/WJFK4O (accessed on 13 May 2021).
